# Photon specific absorbed fractions for the ICRP family of mesh-type reference computational phantoms

**DOI:** 10.1088/1361-6560/ae94db

**Published:** 2026-08-24

**Authors:** Wyatt W Smither, Chansoo Choi, Bangho Shin, Derek W Jokisch, Emmanuel M Mate-Kole, Shaheen A Dewji, Wesley E Bolch

**Affiliations:** 1 J Crayton Pruitt Family Department of Biomedical Engineering, University of Florida, Gainesville, FL, United States of America; 2 Francis Marion University, Florence, SC, United States of America; 3 The Georgia Institute of Technology, George W Woodruff School of Mechanical Eng., Atlanta, GA, United States of America

**Keywords:** ICRP reference computational phantoms, photon specific absorbed fractions, internal dosimetry

## Abstract

*Objective.* To provide photon specific absorbed fractions (SAFs), which quantify the fraction of emitted energy absorbed in target tissues and form the basis for internal dose coefficients, using mesh-type reference computational phantoms (MRCPs). *Approach.* The International Commission on Radiological Protection (ICRP) has recently published MRCPs in Publications 145 and 156 for adult and pediatric reference individuals, respectively. These phantoms supersede the voxel-type reference computational phantoms used in ICRP Publications 133 and 155, overcoming key limitations such as the inability to explicitly define micrometer-scale radiosensitive target tissues, the reliance on supplemental stylized models, and known anatomical inaccuracies such as the lack of full coverage of trabecular spongiosa by cortical bone within the phantom’s skeleton. Photon SAFs were computed using the Particle and Heavy Ion Transport code System Monte Carlo code for all 12 ICRP MRCPs, comprising male and female phantoms at each of six ages, covering 86 source regions and 62 target regions as defined within the ICRP internal dosimetric framework. *Main Results.* The MRCP-based photon SAF dataset demonstrates agreement with ICRP Publications 133 and 155 for most of the crossfire geometries and for the lowest photon energy self-absorption geometries. For photon energies exceeding 30 keV, differences are observed which are consistent with limitations associated with the ICRP reference organ mass scaling approach. SAF differences are also observed due to low-energy extrapolation methods, anatomical improvements including explicit modeling of thin tissue layers, and updated photon dose response functions for the skeletal targets of active marrow and bone endosteum. The datasets exhibit clear age-dependent trends, with younger reference individuals typically yielding higher SAF values. *Significance.* This study provides complete photon SAF datasets spanning all ICRP MRCPs with expanded source-target combinations, improved dosimetric accuracy, all within a unified computational framework to support the calculation of new reference dose coefficients for both tissue-specific equivalent dose and effective dose.

## 1. Introduction

Individuals may be exposed to internal sources of radiation through a variety of medical, occupational, and environmental scenarios. Within the internal dosimetry framework outlined by the International Commission on Radiological Protection (ICRP), such exposures result from the intake or introduction of radionuclides into the body via defined pathways, including the administration of radiopharmaceuticals to medical patients for diagnostic and therapeutic purposes, inhalation of aerosolized radionuclides in occupational or environmental settings, ingestion of radioactive materials through contaminated food or water, and the incorporation of radionuclides through wound contamination or absorption through damaged skin (ICRP 1991, 2007). Following intake, radionuclides may deposit in specific organs and tissues and undergo time-dependent biokinetic transport and retention as described by ICRP systemic, alimentary tract, and respiratory tract models (ICRP 1994, 2006, 2015, 2016b, 2017, 2019, 2022, 2024b). The resulting emissions deliver radiation dose to radiosensitive target regions. Accurate internal dosimetry methods are desirable to estimate organ absorbed and effective doses in accordance with ICRP radiological protection recommendations (ICRP 2007, 2016a, 2017). The quantification of the mean absorbed dose to a target tissue after the intake of a given radionuclide can be computed by:

\begin{equation*}D\left( {{r_{\mathrm{T}}},{T_{\mathrm{D}}}} \right) = \mathop {\sum\limits}\limits_{{r_{\mathrm{S}}}} \mathop \int \limits_0^{{T_{\mathrm{D}}}} A\left( {{r_{\mathrm{S}}},t} \right)S\left( {{r_{\mathrm{T}}} \leftarrow {r_{\mathrm{S}}},t} \right){\mathrm{d}}t\end{equation*}

where *D*(*r*_T_, *T*_D_) is the mean absorbed dose to target tissue ${r_{\mathrm{T}}}$ from radioactive material uniformly distributed within source tissue ${r_{\mathrm{S}}}$ over a dose-integration period of ${T_{\mathrm{D}}}$; $A\left( {{r_{\mathrm{S}}},t} \right)$ is the time-dependent activity of the radionuclide in source tissue ${r_{\mathrm{S}}}$; and $S\left( {{r_{\mathrm{T}}} \leftarrow {r_{\mathrm{S}}},t} \right)$ is the radionuclide-specific absorbed dose in target tissue ${r_{\mathrm{T}}}$ per nuclear transformation in source tissue ${r_{\mathrm{S}}}$. The *S* value (or *S* coefficient), $S\left( {{r_{\mathrm{T}}} \leftarrow {r_{\mathrm{S}}},t} \right)$, encapsulates the radiation emission characteristics of a given radionuclide and the anatomical geometry of the source-target pair and is assumed to be time-independent, $S\left( {{r_{\mathrm{T}}} \leftarrow {r_{\mathrm{S}}}} \right)$, for adults and time-dependent, $S\left( {{r_{\mathrm{T}}} \leftarrow {r_{\mathrm{S}}},t} \right),$ for younger individuals over *T*_D_, consistent with the ICRP formalism. The *S* value can be computed as:

\begin{equation*}S\left( {{r_{\mathrm{T}}} \leftarrow {r_{\mathrm{S}}},t} \right) = \sum\limits_i {E_i}{Y_i}{{\Phi }}\left( {{r_{\mathrm{T}}} \leftarrow {r_{\mathrm{S}}},{E_i}} \right)\end{equation*}

where ${E_i}$ is the energy of nuclear transformation of index *i* for a given radionuclide, ${Y_i}$ is the corresponding yield of nuclear transformation of index *i* for a given radionuclide, and ${{\Phi }}\left( {{r_{\mathrm{T}}} \leftarrow {r_{\mathrm{S}}},{E_i}} \right)$ is the specific absorbed fraction (SAF) to target tissue ${r_{\mathrm{T}}}$ from source tissue ${r_{\mathrm{S}}}$ for energy ${E_i}$. The SAF can be computed by

\begin{equation*}{{\Phi }}\left( {{r_{\mathrm{T}}} \leftarrow {r_{\mathrm{S}}},{E_i}} \right) = \frac{{\phi \left( {{r_T} \leftarrow {r_{\mathrm{S}}},{E_i}} \right)}}{{m\left( {{r_{\mathrm{T}}}} \right)}}\end{equation*}

where $\phi \left( {{r_{\mathrm{T}}} \leftarrow {r_{\mathrm{S}}},{E_i}} \right)$ is the absorbed fraction (AF) which is the fraction of energy emitted of energy ${E_i}$ in source tissue ${r_{\mathrm{S}}}$ that is deposited in target tissue ${r_{\mathrm{T}}}$, and $m\left( {{r_{\mathrm{T}}}} \right)$ is the mass of target tissue ${r_{\mathrm{T}}}$.

ICRP Publications 133 and 155 provide reference SAF datasets for photons, electrons, alpha particles, and fission-spectrum neutrons from internally emitted sources, with most SAF values derived from Monte Carlo radiation transport simulations using the ICRP reference adult and pediatric voxel-type reference computational phantoms (VRCPs) described in ICRP Publications 110 and 143, respectively (ICRP 2009, 2016a, 2020a, 2023). Despite the significant advances represented by these datasets, several limitations remain. For example, the spatial resolution and inherent geometric limitations of voxel phantoms prevent the explicit representation of micrometer-scale source and target regions, such as those in the respiratory and alimentary tracts. Consequently, SAFs for charged particles in these tissues were derived using the stylized models described in ICRP Publications 66 and 100 (ICRP 1994, 2006) rather than directly within the whole-body phantoms. Consequently, the reference SAF datasets rely on a combination of voxel phantoms and supplemental stylized models rather than a single anatomically consistent computational phantom. In addition, the reference organ masses correspond to the organ parenchyma only and do not include the blood contained within those organs (ICRP 2009, 2020a). To account for the missing organ blood content, the SAFs for self-irradiation cases were scaled using the corresponding blood-inclusive target organ masses (ICRP 2016a, 2023).

The ICRP has addressed the shortcomings of the VRCPs through the development of mesh-type reference computational phantoms (MRCPs), published for adult and pediatric reference individuals in ICRP Publications 145 and 156, respectively (ICRP 2020b, 2024a). The MRCPs were developed by converting the VRCPs into high-fidelity mesh phantoms while incorporating anatomically realistic organ boundaries and explicitly modeling micrometer-thick source and radiosensitive target tissues within the skin, eye lens, urinary bladder, and alimentary and respiratory tracts. These features enable the calculation of SAFs within a single anatomically consistent whole-body phantom, thereby eliminating the need for supplemental stylized models used in ICRP Publications 133 and 155. Acknowledging these advancements, a recent study (El Ghalbzouri *et al* 2026) has generated photon and electron SAFs using the adult male MRCP. However, that study was restricted to selected photon energies and a limited number of source-target region combinations, and a comprehensive reference dataset has yet to be established.

The purpose of this study is to update reference photon SAFs provided by Schwarz *et al* (2021) and the ICRP (2016a, 2023) using the family of MRCPs recently published by the ICRP (2020b, 2024a). The use of MRCPs has enabled expanded source-target combinations in a whole-body phantom, improved dosimetric accuracy through the elimination of limitations associated with VRCPs, updated SAFs using human respiratory tract model (HRTM) and human alimentary tract model (HATM) incorporation into the whole-body phantom, and implementation of updated photon fluence-to-dose response functions (DRFs) for skeletal tissues. Updated smoothing and low-energy SAF extrapolation methods were also incorporated as independent improvements.

## 2. Materials and methods

### 2.1. Description of ICRP MRCPs

The ICRP has published adult and pediatric MRCPs in Publications 145 and 156, respectively, representing substantial updates to their VRCP counterparts from Publications 110 and 143. These MRCPs were created by converting the voxel phantoms into high-quality mesh format, integrating supplementary stylized models, and adding anatomically relevant tissue layers containing cells considered to be at risk of radiation-induced cancers. Notably, the MRCPs explicitly define micrometer-scale target layers in the alimentary and respiratory tracts, skin, and urinary bladder, in addition to all source and target organs and tissues required for the calculation of effective dose. The MRCPs also incorporate several anatomical corrections relative to the VRCPs, including explicit inclusion of intra-organ blood content, a substantially revised skeletal system where anatomical refinements to individual skeletal tissues were made (Choi *et al* 2021), updated colon and small intestine models with anatomically realistic wall thicknesses and target cell layers (Choi *et al* 2021), refined thyroid geometry (Yeom *et al* 2022), updated lymphatic node distributions, improved modeling of the extrathoracic (ET) regions of the respiratory tract (Choi *et al* 2023), detailed eye models (Han *et al* 2021), and improved representations of teeth (Shin *et al* 2021), oral structures, and muscle tissue distributions.

The mesh-type format provides several technical advantages central to this work. Tetrahedral meshes eliminate the stair-step artifacts inherent to voxelized structures, enabling smooth and anatomically realistic organ boundaries. Additionally, tetrahedral meshes have been shown to reduce radiation transport simulation times by up to a factor of three compared to VRCPs (Yeom *et al* 2019).

### 2.2. SAF calculations for photons

In the present study, photon SAFs were calculated for all defined source-target region combinations listed in tables 1 and 2 using the ICRP adult and pediatric MRCPs described in ICRP Publications 145 and 156. SAFs were generated for ICRP reference male and female individuals at ages newborn, 1 y, 5 y, 10 y, 15 y, and adult and were intended to update and extend the reference photon SAF datasets previously reported in ICRP Publications 133 and 155. Calculations were performed using anatomically realistic source and target definitions, including micrometer-scale target tissues and expanded source and target regions not used in earlier works.

**Table 1.** Source regions for all ages and whether they are included in ICRP Publication 133, ICRP Publication 155, or the present study.

Source region	Acronym	ICRP-133	ICRP-155	Present study
Oral cavity	O-cavity	**✓**	**✓**	**✓**
Oral mucosa	O-mucosa	**✓**	**✓**	**✓**
Teeth surface	Teeth-S	**✓**	**✓**	**✓**
Teeth volume	Teeth-V	**✓**	**✓**	**✓**
Tongue	Tongue	**✓**	**✓**	**✓**
Tonsils	Tonsils	**✓**	**✓**	**✓**
Oesophagus fast	Oesophag-f	**✓**	**✓**	**✓**
Oesophagus slow	Oesophag-s	**✓**	**✓**	**✓**
Oesophagus	Oesophag-w	**✓**	**✓**	**✓**
Stomach contents	St-cont	**✓**	**✓**	**✓**
Stomach wall	St-wall	**✓**	**✓**	**✓**
Stomach mucosa	St-mucosa	**✓**	**✓**	**✓**
Small intestine contents	SI-cont	**✓**	**✓**	**✓**
Small intestine villi	SI-villi	**✓**	**✓**	**✓**
Small intestine wall	SI-wall	**✓**	**✓**	**✓**
Small intestine mucosa	SI-mucosa	**✓**	**✓**	**✓**
Right colon contents	RC-cont	**✓**	**✓**	**✓**
Right colon wall	RC-wall	**✓**	**✓**	**✓**
Right colon mucosa	RC-mucosa	**✓**	**✓**	**✓**
Left colon contents	LC-cont	**✓**	**✓**	**✓**
Left colon wall	LC-wall	**✓**	**✓**	**✓**
Left colon mucosa	LC-mucosa	**✓**	**✓**	**✓**
Rectosigmoid colon contents	RS-cont	**✓**	**✓**	**✓**
Rectosigmoid colon wall	RS-wall	**✓**	**✓**	**✓**
Rectosigmoid colon mucosa	RS-mucosa	**✓**	**✓**	**✓**
Anterior nasal passage surface	ET_1_-sur	**✓**	**✓**	**✓**
Posterior nasal passage surface	ET_2_-sur	**✓**	**✓**	**✓**
Anterior nasal passage wall	ET_1_-wall	**✓**	**✓**	**✓**
Posterior nasal passage wall	ET_2_-wall	**✓**	**✓**	**✓**
Posterior nasal passage bound region	ET_2_-bnd	**✓**	**✓**	**✓**
Posterior nasal passage sequestered region	ET_2_-seq	**✓**	**✓**	**✓**
Extrathoracic lymph nodes	LN-ET	**✓**	**✓**	**✓**
Bronchi	Bronchi	**✓**	**✓**	**✓**
Bronchi bound region	Bronchi-b	**✓**	**✓**	**✓**
Bronchi sequestered region	Bronchi-q	**✓**	**✓**	**✓**
Bronchiole	Brchiole	**✓**	**✓**	**✓**
Bronchiolar bound region	Brchiole-b	**✓**	**✓**	**✓**
Bronchiolar sequestered region	Brchiole-q	**✓**	**✓**	**✓**
Alveolar interstitium	ALV	**✓**	**✓**	**✓**
Lung tissue	Lung-Tis	**✓**	**✓**	**✓**
Respiratory tract air	RT-Air	**✓**	**✓**	**✓**
Thoracic lymph nodes	LN-Th	**✓**	**✓**	**✓**
Right adrenal + left adrenal	Adrenals	**✓**	**✓**	**✓**
Major blood vessels	MBV	**✕**	**✕**	**✓**
Blood in heart	Ht-cont	**✕**	**✕**	**✓**
Total blood	Blood	**✓**	**✓**	**✓**
Cortical bone surface	C-bone-S	**✓**	**✓**	**✓**
Cortical bone volume	C-bone-V	**✓**	**✓**	**✓**
Trabecular bone surface	T-bone-S	**✓**	**✓**	**✓**
Trabecular bone volume	T-bone-V	**✓**	**✓**	**✓**
Cortical bone marrow	C-marrow	**✓**	**✓**	**✓**
Red (active) bone marrow	R-marrow	**✓**	**✓**	**✓**
Trabecular bone marrow	T-marrow	**✓**	**✓**	**✓**
Yellow (inactive) bone marrow	Y-marrow	**✓**	**✓**	**✓**
Brain	Brain	**✓**	**✓**	**✓**
Breast adipose + breast glandular	Breast	**✓**	**✓**	**✓**
Lens of eye	Eye-lens	**✓**	**✓**	**✓**
Gallbladder	GB-wall	**✓**	**✓**	**✓**
Gallbladder contents	GB-cont	**✓**	**✓**	**✓**
Heart	Ht-wall	**✓**	**✓**	**✓**
Right kidney cortex	RKidney-C	**✕**	**✕**	**✓**
Right kidney medulla	RKidney-M	**✕**	**✕**	**✓**
Right kidney pelvis	RKidney-P	**✕**	**✕**	**✓**
Left kidney cortex	LKidney-C	**✕**	**✕**	**✓**
Left kidney medulla	LKidney-M	**✕**	**✕**	**✓**
Left kidney pelvis	LKidney-P	**✕**	**✕**	**✓**
Right kidney + left kidney	Kidneys	**✓**	**✓**	**✓**
Liver	Liver	**✓**	**✓**	**✓**
Systemic lymph nodes	LN-Sys	**✓**	**✓**	**✓**
Muscle	Muscle	**✓**	**✓**	**✓**
Right ovary + left ovary	Ovaries	**✓**	**✓**	**✓**
Pancreas	Pancreas	**✓**	**✓**	**✓**
Pituitary gland	P-gland	**✓**	**✓**	**✓**
Prostate	Prostate	**✓**	**✓**	**✓**
Salivary glands	S-glands	**✓**	**✓**	**✓**
Skin	Skin	**✓**	**✓**	**✓**
Spleen	Spleen	**✓**	**✓**	**✓**
Testes	Testes	**✓**	**✓**	**✓**
Thymus	Thymus	**✓**	**✓**	**✓**
Thyroid	Thyroid	**✓**	**✓**	**✓**
Ureters	Ureters	**✓**	**✓**	**✓**
Urinary bladder	UB-wall	**✓**	**✓**	**✓**
Urinary bladder content	UB-cont	**✓**	**✓**	**✓**
Uterus/cervix	Uterus	**✓**	**✓**	**✓**
Adipose/residual tissue	Adipose	**✓**	**✓**	**✓**
Cartilage	Cartilage	**✓**	**✓**	**✓**

**Table 2.** Target regions for all ages and whether they are included in ICRP Publication 133, ICRP Publication 155, or the present study.

Target region	Acronym	ICRP-133	ICRP-155	Present study
Oral mucosa	O-mucosa	**✓**	**✓**	**✓**
Oesophagus wall	Oesophagus	**✓**	**✓**	**✓**
Oesophagus basal cells	Oesophag-bas	**✕**	**✕**	**✓**
Stomach wall	St-wall	**✕**	**✕**	**✓**
Stomach stem cells	St-stem	**✓**	**✓**	**✓**
Small intestine wall	SI-wall	**✕**	**✕**	**✓**
Small intestine stem cells	SI-stem	**✓**	**✓**	**✓**
Colon wall	Colon-wall	**✕**	**✕**	**✓**
Colon stem cells	Colon-stem	**✕**	**✕**	**✓**
Right colon wall (ascending + right transverse)	RC-wall	**✕**	**✕**	**✓**
Right colon stem cells (ascending + right transverse)	RC-stem	**✓**	**✓**	**✓**
Left colon wall (left transverse + descending)	LC-wall	**✕**	**✕**	**✓**
Left colon stem cells (left transverse + descending)	LC-stem	**✓**	**✓**	**✓**
Rectosigmoid colon wall (sigmoid + rectum)	RS-wall	**✕**	**✕**	**✓**
Rectosigmoid colon stem cells (sigmoid + rectum)	RS-stem	**✓**	**✓**	**✓**
Anterior nasal passage wall	ET_1_-wall	**✕**	**✕**	**✓**
Posterior nasal passage wall	ET_2_-wall	**✕**	**✕**	**✓**
Anterior nasal passage basal cells	ET_1_-bas	**✓**	**✓**	**✓**
Posterior nasal passage basal cells	ET_2_-bas	**✓**	**✓**	**✓**
Extrathoracic lymph nodes	LN-ET	**✓**	**✓**	**✓**
Bronchi basal cells	Bronch-bas (Gen 1)	**✓**	**✓**	**✓**
Bronchi sequestered region	Bronch-sec (Gen 1)	**✓**	**✓**	**✓**
Bronchiolar secretory cells	Brchiol-sec	**✓**	**✓**	**✓**
Alveolar interstitium	AI	**✓**	**✓**	**✓**
Thoracic lymph nodes	LN-Th	**✓**	**✓**	**✓**
Brain	Brain	**✓**	**✓**	**✓**
Lens of eye	Eye-lens	**✓**	**✓**	**✓**
Pituitary gland	P-gland	**✓**	**✓**	**✓**
Tongue	Tongue	**✓**	**✓**	**✓**
Tonsils	Tonsils	**✓**	**✓**	**✓**
Salivary glands	S-glands	**✓**	**✓**	**✓**
Thyroid	Thyroid	**✓**	**✓**	**✓**
Breast adipose + breast glandular	Breast	**✓**	**✓**	**✓**
Thymus	Thymus	**✓**	**✓**	**✓**
Heart wall	Ht-wall	**✓**	**✓**	**✓**
Right adrenal + left adrenal	Adrenals	**✓**	**✓**	**✓**
Liver	Liver	**✓**	**✓**	**✓**
Pancreas	Pancreas	**✓**	**✓**	**✓**
Right kidney + left kidney	Kidneys	**✓**	**✓**	**✓**
Right kidney cortex	RKidney-C	**✕**	**✕**	**✓**
Right kidney medulla	RKidney-M	**✕**	**✕**	**✓**
Right kidney pelvis	RKidney-P	**✕**	**✕**	**✓**
Left kidney cortex	LKidney-C	**✕**	**✕**	**✓**
Left kidney medulla	LKidney-M	**✕**	**✕**	**✓**
Left kidney pelvis	LKidney-P	**✕**	**✕**	**✓**
Spleen	Spleen	**✓**	**✓**	**✓**
Gallbladder wall	GB-wall	**✓**	**✓**	**✓**
Ureters	Ureters	**✓**	**✓**	**✓**
Urinary bladder wall	UB-wall	**✓**	**✓**	**✓**
Urinary bladder basal cells	UB-bas	**✕**	**✕**	**✓**
Systemic lymph nodes	LN-Sys	**✓**	**✓**	**✓**
Skin	Skin	**✓**	**✓**	**✓**
Skin basal cells	Skin-bas	**✕**	**✕**	**✓**
Adipose/residual tissue	Adipose	**✓**	**✓**	**✓**
Muscle	Muscle	**✓**	**✓**	**✓**
Spinal cord	Sp-cord	**✕**	**✕**	**✓**
Red (active) bone marrow	R-marrow	**✓**	**✓**	**✓**
50-*μ*m endosteal region	Endost-BS	**✓**	**✓**	**✓**
Right ovary + left ovary	Ovaries	**✓**	**✓**	**✓**
Uterus/cervix	Uterus	**✓**	**✓**	**✓**
Prostate	Prostate	**✓**	**✓**	**✓**
Testes	Testes	**✓**	**✓**	**✓**

Unlike the previous ICRP VRCPs, which excluded intra-organ blood from reference organ masses and volumes, the ICRP MRCP volumes are defined inclusive of both parenchyma and intra-organ blood. Consequently, while previous datasets (ICRP 2016a, 2023, Schwarz *et al* 2021) required a post-simulation mass-scaling approach to account for blood content, the SAFs presented in this study are computed directly from the reference masses inclusive of intra-organ blood, eliminating the need for scaling.

Tables 1 and 2 detail the source and target regions, respectively, defined in ICRP Publications 145 and 156 provided alongside their corresponding abbreviations and a comparative indication of their inclusion in the ICRP Publication 133 and 155 datasets versus the present work. For source regions composed of multiple anatomical sub-structures, each constituent was modeled as a discrete geometry weighted by its reference volume; for example, the ‘kidneys’ source region was defined as a composite of the left and right cortex, medulla, and pelvis. For skeletal source regions, a surrogate approach had to be implemented where volume fractions for each individual skeletal site were used within the Monte Carlo simulation; for example, a total-body ‘R-marrow’ source was achieved by weighting each individual bone site by its fractional contribution to the total red bone marrow. In contrast, the micrometer-scale source and target layers of the alimentary, integumentary, and urinary systems are explicitly defined within the MRCPs and were used directly in the present calculations without the need to use surrogate tissues for both self-absorption and crossfire cases across the entire energy range. Notably, SAFs for these distributed sources were computed directly within a Monte Carlo simulation. It should be noted that teeth surfaces and yellow (inactive) bone marrow are excluded as source regions in the newborn phantoms, consistent with neonatal physiological development.

Recently, Shin *et al* (2025) have published updated SAFs and *S* values for intra-HRTM geometries, which provides highly detailed respiratory tract structures and airways represented using tetrahedral mesh and constructive solid geometry (CSG) formats. These datasets were generated using the adult and pediatric ICRP MRCPs. SAFs for photons, electrons, and alpha particles were calculated across relevant energy ranges using Geant4 Monte Carlo radiation transport simulations combined with nuclear decay data from ICRP Publication 107 to derive radionuclide *S* values for inhalation exposures. These SAF and *S* value datasets explicitly accounted for micrometer-scale source and target regions and, for the first time, included a full set of HRTM geometries. The resulting SAFs and *S* values exhibited pronounced age dependence and were typically larger than corresponding values reported in ICRP Publication 133 and 155 due to differences in target masses and detailed airway branching. The photon SAF values reported by Shin *et al* were appended, as published, to the tabulated datasets of the present study; accordingly, no new Monte Carlo simulations were performed for these cases. As a lung tissue (Lung-Tis) source was not included in the Shin *et al* dataset, new Geant4 Monte Carlo simulations for bronchial wall and bronchiolar wall sources were conducted in the present study following their established methodology.

**Figure 1.**
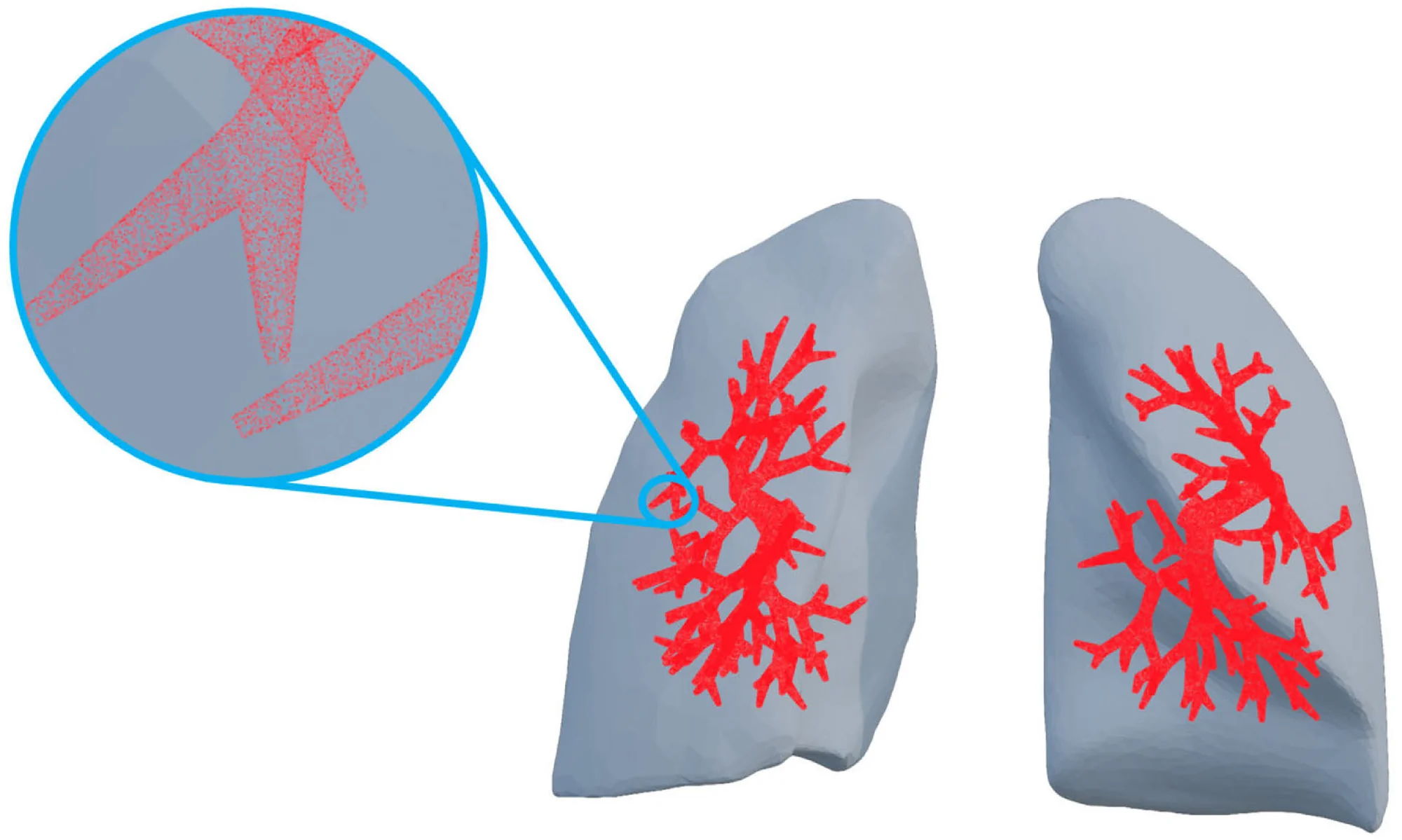
Generated source points in the fast and slow tissue layers within generations 2–8 of the bronchial region for the 5 yr old female MRCP.

To evaluate crossfire doses from HRTM sources (e.g. from bronchial sources to non-HRTM targets like the brain), source-point distributions were generated from the CSG-format airway models for specific source regions and integrated into the homogenized lung models of the MRCPs. For each source region, the CSG-defined volume was populated with an even, discrete distribution of points, and the *x, y, z* coordinates of these points were supplied to Monte Carlo input files as source positions. These source-point distribution files therefore contained only the spatial coordinates of the source points; the emission direction of each primary photon was not stored but was instead sampled isotopically at run time in the radiation transport simulation. Discrete source-point files were generated in this manner for the lung tissue, alveolar interstitium, respiratory tract air, and each bronchial and bronchiolar region. For instance, figure 1 illustrates the source-point distribution for the slow and fast tissue layers within generations 2–8 of the bronchial region for the 5 yr old female MRCP.

### 2.3. Monte Carlo radiation transport simulations

To generate the photon SAF datasets, the Particle and Heavy Ion Transport code System (PHITS) version 3.35 (Sato *et al* 2024), a benchmarked (Iwamoto *et al* 2022) general-purpose Monte Carlo particle transport simulation code, was used with the EGS5 cross section library for photon and electron transport (Hirayama *et al* 2016). Photon source particles were run on a logarithmic scale from 10 keV to 10 MeV, consistent with previous studies (ICRP 2016a, 2023, Schwarz *et al* 2021). Absorbed doses were calculated in units of Gy per source using the *T*-deposit tally in PHITS for the target regions listed in table 2. To derive SAFs, the PHITS dose tallies were converted from joules to MeV and normalized to the initial particle energy. The resulting SAFs were then expressed in units of inverse mass (kg^−1^). The radiation transport simulations were run utilizing the multithreading of 24 cores on the University of Florida’s supercomputer, HiPerGator, which has 19 200 AMD EPYC 9655P Turin 2.5 GHz (4.5 GHz Boost) cores. Due to there being over 25 000 individual radiation transport simulations required for this work, efforts were made to minimize computation time while maintaining low statistical uncertainties in the tallied results. A tiered particle-history approach was implemented: photon sources below 80 keV were simulated using 10^9^ particles; sources between 100 keV and 800 keV used 5 × 10^8^ particles; and sources between 1 MeV and 10 MeV used 10^8^ particles. Photon cutoff energies were uniformly set to 1 keV, while electron and positron cutoff energies were set to 1 keV for photon source energies below 1 MeV and 10 keV for energies at or above 1 MeV. No variance reduction or tally filtering/denoising techniques were employed. Updated fluence-to-DRFs for red (active) bone marrow and bone endosteum were implemented within the PHITS simulation where the DRF as a function of traversing particle energy was log–log interpolated and applied to report SAFs for red bone marrow and the bone endosteum (ICRP 2025). These updated DRFs correct a small number of low-energy cases where the previous DRFs resulted in a dose reduction in red bone marrow relative to secondary electron equilibrium, which is a physically inconsistent result as the higher density of mineral bone cannot produce a deficit of secondary electrons in the adjacent marrow.

### 2.4. Data processing and smoothing

SAF data analysis, processing, and smoothing were performed using MATLAB^4^4MATLAB, version R2025b, The MathWorks Inc., Natick, MA, USA.. Relative errors for each organ tallied in PHITS were saved. Any nonzero SAF value with a relative error exceeding 0.50 was set to zero, since a relative error above this threshold has historically been regarded as indicative of a statistically unreliable or meaningless tally (Shultis and Bahadori 2024), and, at such levels, contributes negligibly to the total dose at a given initial particle energy. To ensure the nonzero SAF values formed a single continuous sequence, any zero embedded within the nonzero values was treated as a gap, and all values preceding the last such gap were set to zero. For example, [0 0 # 0 # # # #] became [0 0 0 0 # # # #]. This occurred primarily for source-target combinations that were spatially far apart, where the low number of scored events produced large relative errors that were subsequently zeroed out by the 0.50 threshold while the SAF at the previous energy point was not.

For the remaining valid, nonzero SAF data points with relative errors less than 0.50, a weighted Gaussian process regression (GPR) was performed in log–log space. Let x>0 denote the initial photon energies and y>0 the SAF values. These were transformed to logarithmic space as

\begin{equation*}u = {\log _{10}}\left( x \right){\text{ }}v = {\log _{10}}\left( y \right)\end{equation*}

and the regression problem was formulated as

\begin{equation*}v\left( u \right) = f\left( u \right) + \varepsilon \end{equation*}

where $f\left( u \right)$ is an unknown smooth function and ε represents observation noise. The function $f\left( u \right)$ was modeled as a Gaussian process prior

\begin{equation*}f\left(u\right) \sim {\mathrm{GP}}\left(0,k\left(u,u{^{^{\prime}}}\right)\right)\end{equation*}

with a squared-exponential (radial basis) kernel

\begin{equation*}k\left( {u,u^{\prime}} \right) = \sigma _f^2{\mathrm{exp}}\left[ { - \frac{{{{\left( {u - u^{\prime}} \right)}^2}}}{{2{l^2}{\text{ }}}}} \right]{\text{ }}\end{equation*}

where $\sigma _f^2$ is the signal variance, and *l* is the characteristic length scale controlling smoothness.

To account for heteroscedastic uncertainty, the relative error in linear space was converted to a standard deviation in log-space as

\begin{equation*}{\sigma _{{\mathrm{log,}}i}} = \frac{{{\mathrm{reler}}{{\mathrm{r}}_i}}}{{{\mathrm{ln}}\left( {10} \right)}}\end{equation*}

and the corresponding weight for each data point was defined by

\begin{equation*}{w_i} = \frac{1}{{\sigma _{{\mathrm{log}},i{ }}^2}}\end{equation*}

which was normalized by the maximum weight to improve numerical stability.

The GPR posterior mean predictions at the training points were then transformed back to linear space,

\begin{equation*}\hat y = {10^{\hat v}}\end{equation*}

and used to reconstruct smoothed SAF values, preserving points with low Monte Carlo relative errors within their uncertainty bands and maintaining realistic trends while accounting for pointwise measurement uncertainty.

### 2.5. Limiting values and low-energy extrapolation

SAF values were extrapolated to their limiting value at 0 MeV. ICRP Publications 133 and 155 (ICRP 2016a, 2023) provided low-energy limiting values for SAF values shown here in equation (11) through (16). The limiting expressions for completely overlapping (source and target are the same) and non-overlapping (source and target are different) source-target configurations are given by

\begin{equation*}\mathop {\lim }\limits_{E \to 0} {{\Phi }}\left( {{r_{\mathrm{T}}} \leftarrow {r_{\mathrm{S}}},{E_0}} \right) = \{ \begin{array}{*{20}{c}} {\frac{1}{{{m_{\mathrm{T}}}}},{\text{ if }}{r_{\mathrm{S}}} = {r_{\mathrm{T}}}} \\ {0,{\text{ if }}{r_{\mathrm{S}}} \ne {r_{\mathrm{T}}}} \end{array}{ }\end{equation*}

where ${m_{\mathrm{T}}}$ is the mass in kilograms of the target tissue. For blood sources, the limiting SAF can be computed by

\begin{equation*}\mathop {\lim }\limits_{E \to 0} {{\Phi }}\left( {{r_{\mathrm{T}}} \leftarrow {\mathrm{Blood}},E} \right) = \frac{{{f_{{r_{\mathrm{T}}}}}}}{{{m_{\mathrm{T}}}}}\end{equation*}

where ${f_{{r_{\mathrm{T}}}}}{ }$ is the fraction of the body’s total blood in target tissue ${r_{\mathrm{T}}}.{ }$ Similar to this blood source, in which a portion of the source organ resides within the target region, certain source-target combinations, such as those in the alimentary tract, exhibit partial overlap. For these cases, the limiting AF is equal to the fraction of the source region’s volume that is designated as the target volume:

\begin{equation*}\mathop {\lim }\limits_{E \to 0} {{\Phi }}\left( {{r_{\mathrm{T}}} \leftarrow {r_{\mathrm{S}}}} \right) = \frac{{\frac{{{V_{{\mathrm{S}}{{\mathop \cap \nolimits}}{\mathrm{T}}}}}}{{{V_{\mathrm{S}}}}}}}{{{m_{\mathrm{T}}}}}\end{equation*}

where ${V_{{\mathrm{S}}{{\mathop \cap \nolimits}}{\mathrm{T}}}}$ is the volume of the source tissue overlapping the target tissue, and ${V_{\mathrm{S}}}$ is the total volume of the source tissue. If the densities of the source and target tissues are identical, the volume ratio can be replaced with mass ratio of these regions.

The skeleton in the ICRP MRCPs is subdivided into cortical bone, spongiosa, and medullary cavities. As noted, fluence-to-DRFs were required to compute absorbed doses to red (active) bone marrow and bone endosteum, since explicit microscopic bone structure models are not represented within the MRCPs. Consequently, special formulations were used to determine limiting SAFs for certain source-target combinations within the skeleton. For photon energies approaching zero, the following limiting values for marrow sources to bone endosteum targets can be computed by

\begin{equation*}\mathop {\lim }\limits_{E \to 0} {{\Phi }}\left({\mathrm{Endost-BS}} \leftarrow {\mathrm{Marrow}},{\text{ }}E\right) = \frac{{\frac{{{V_{{\mathrm{Endost-BS}}}}}}{{{V_{{\mathrm{Marrow}}}}}}}}{{{m_{{\mathrm{Endost-BS}}}}}} = \frac{{\frac{{{m_{{\mathrm{Endost-BS}}}}}}{{{m_{{\mathrm{Marrow}}}}}}}}{{{m_{{\mathrm{Endost-BS}}}}}} = \frac{1}{{{m_{{\mathrm{Marrow}}}}}}\end{equation*}

where the endosteal bone surface (Endost-BS) is entirely contained within the marrow volume. For trabecular bone surface sources, only half the emitted energy is assumed to be deposited within the endosteal region in the zero-energy limit, since photons are assumed to be equally likely to be emitted in either direction normal to the bone surface. This limiting value is given by

\begin{equation*}\mathop {\lim }\limits_{E \to 0} \left( {{\mathrm{Endost-BS}} \leftarrow {\text{T-bone-S, }}E} \right) = \frac{{\frac{1}{2}}}{{{m_{{\mathrm{Endost-BS}}}}}}.\end{equation*}

Finally, the limiting SAF for activity on the trabecular bone surface irradiating the red marrow can be computed by

\begin{equation*}\mathop {\lim }\limits_{E \to 0} {{{\Phi }}_{{\mathrm{skeleton}}}}\left( {{\text{R-marrow }} \leftarrow {\text{ T-bone-S, }}E} \right) = {\text{ }}\mathop {\mathop \sum \nolimits }\limits_i \frac{{S{A_{{\mathrm{BS}},i{\text{ }}}}}}{{S{A_{{\text{BS, skeleton}}}}}}\frac{{{c_i}}}{{2{m_{{\mathrm{Endost-BS,}}i}}}}\end{equation*}

where $S{A_{{\mathrm{BS}},i{\text{ }}}}$ is the surface area of the trabecular bone surface of skeletal site *i*, $S{A_{{\mathrm{BS}},i{\text{ }}}}$ is the total surface area of all trabecular bone in the skeleton, ${c_i}$ is the cellularity of red bone marrow in site *i*, and ${m_{{\text{Endost - BS,}}i}}$ is the mass of the bone endosteum in bone site *i*.

The approach used in ICRP Publications 133 and 155 to provide SAF values at 1 and 5 keV, described by Schwarz *et al* (2021) and subsequently adopted by the ICRP (ICRP 2023), employed a rejection algorithm based on the maximum SAF and its corresponding statistical uncertainty for each source-target pair, followed by a linear fit in log–log space. In several instances in their work, zero-valued SAFs were replaced with nonzero values to enable fitting of a linear curve (in log–log space), constrained to a limiting value of 10^−12^ kg^−1^ at an energy of 1 eV. This approach also replaced SAF values obtained from Monte Carlo simulations with interpolated values. In the present study, SAFs at 1 and 5 keV were computed using piecewise cubic Hermite interpolating polynomial (PCHIP) interpolation in log–log space, based on the photon SAF values from 10 keV to 10 MeV and the limiting value at 0 MeV. For non-overlapping source-target pairs, the limiting SAF approaches zero as the photon energy approaches 0 MeV, as shown by equation (11). However, because the logarithm of zero is undefined, for numerical convenience, a small nonzero value of 10^−12^ kg^−1^ at 1 eV was used as the lower boundary point for the interpolation.

### 2.6. Quality assurance

Adherence to the ICRP methodological framework necessitates rigorous quality assurance protocols to guarantee the reproducibility and precision of dosimetric quantities derived from Monte Carlo radiation transport simulations. To validate the computational integrity of the current models, a benchmarking study was conducted using the adult male MRCP. Specifically, selected source-target organ combinations were cross-evaluated by one of the coauthors (BS), independent of the primary photon SAF calculations, using Geant4 version 11.01.p02 (Allison *et al* 2016) to verify their consistency against primary values reported by PHITS. This inter-code comparison serves to minimize systematic uncertainties and ensures that the simulated energy deposition remains independent of the specific transport algorithm employed.

## 3. Results and discussion

### 3.1. Photon SAFs

In the present study, we generated comprehensive datasets of photon SAFs for the full family of ICRP MRCPs using the PHITS Monte Carlo code, comprising 5332 source-target combinations across 28 energy points (0–10 MeV), all with Monte Carlo relative errors below 0.50. By employing the MRCPs, this work utilizes in-phantom micrometer-scale radiosensitive targets and eliminates both the stair-step artifacts of voxelized geometries, and the supplemental stylized models previously required to compensate for them, yielding fully consistent, phantom-based datasets. Photon SAFs were computed directly from reference masses inclusive of intra-organ blood, and a pipeline of GPR smoothing with PCHIP interpolation preserved Monte Carlo trends while providing reliable extrapolation down to 0 MeV.

### 3.2. Photon SAF data smoothing

**Figure 2.**
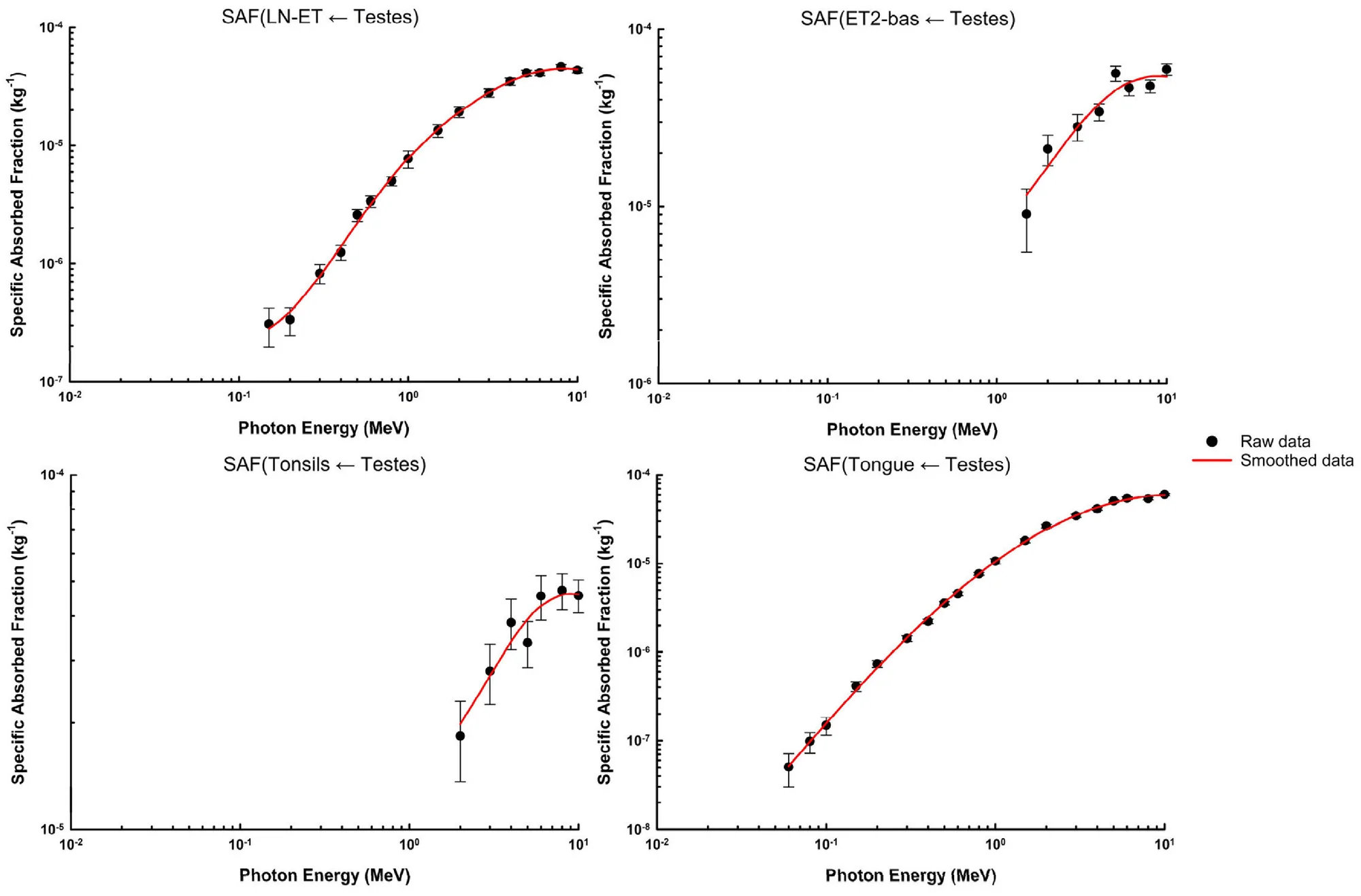
Comparison of raw Monte Carlo-derived specific absorbed fraction (SAF) values (black circles) and GPR-smoothed SAF values (red diamonds) for the adult male MRCP. Results are shown for testes as the source region and ET lymphatic nodes, posterior nasal passage basal cells, tonsils, and tongue as target regions across the photon energy range. Error bars in the raw data represent 1*σ*.

Figure 2 illustrates the results of the GPR smoothing applied to SAFs for the adult male MRCP with the testes as the source region and ET lymphatic nodes, posterior nasal passage basal cells, tonsils, and tongue as target regions. The smoothing algorithm preserves SAF values derived from Monte Carlo simulations when the relative statistical uncertainties are low, while more strongly adjusting the data points with higher relative uncertainties. This approach yields a smoothly varying SAF-energy relationship while retaining the underlying trends of the Monte Carlo results.

### 3.3. Quality assurance

**Figure 3.**
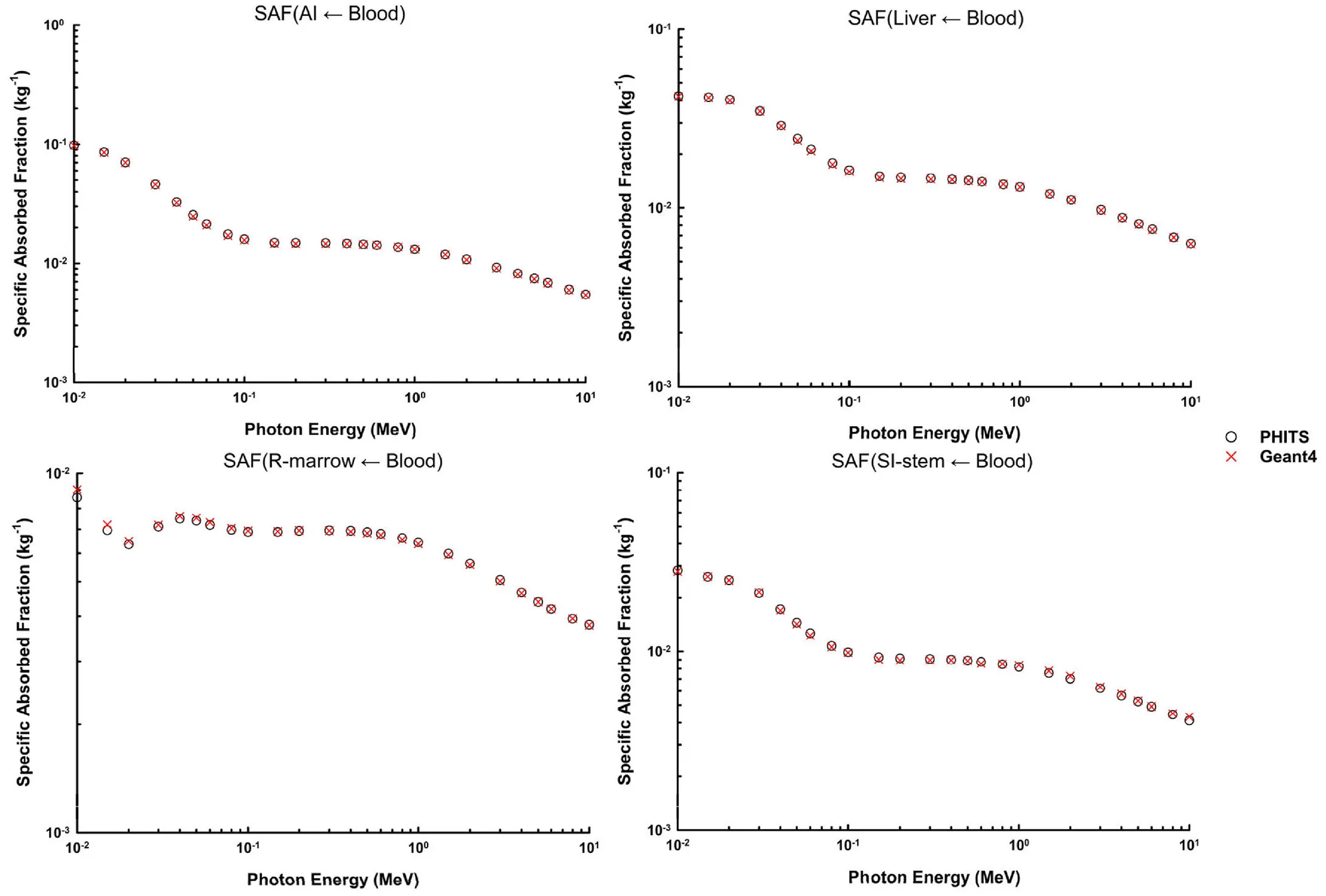
Specific absorbed fractions (SAFs) for blood as a source, and the alveolar interstitium, liver, red (active) bone marrow, and small intestine stem cells as targets for photon exposures calculated with the adult male mesh-type computations phantoms in PHITS (black circles) and Geant4 (red crosses).

**Figure 4.**
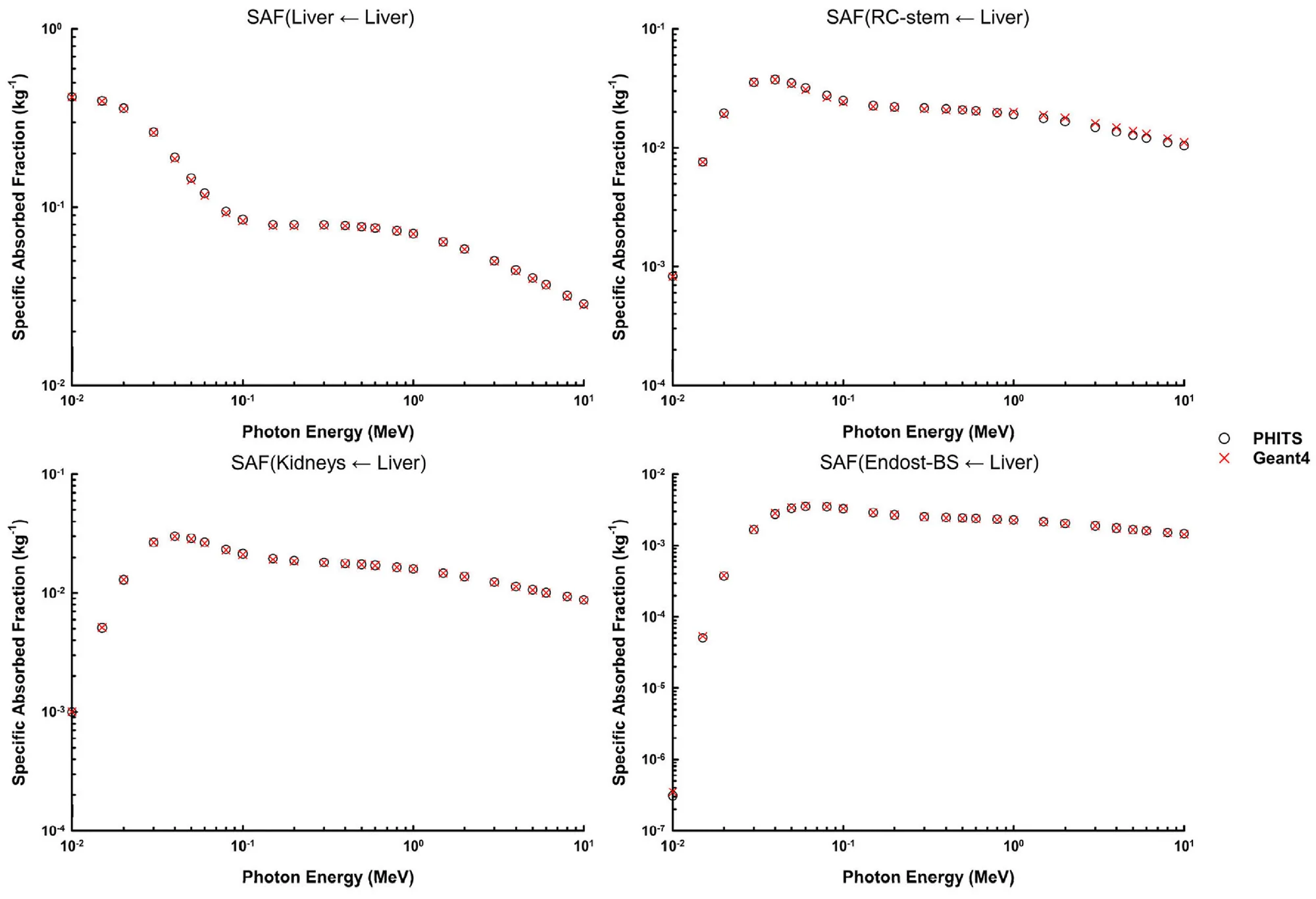
Specific absorbed fractions (SAFs) for liver as a source, and the liver, right colon stem cells, kidneys, and endosteal surface as targets for photon exposures calculated with the adult male mesh-type computations phantoms in PHITS (black circles) and Geant4 (red crosses).

Figures 3 and 4 present unsmoothed photon SAFs for selected source-target combinations in the adult male MRCP calculated using PHITS and independently verified with Geant4. Across the full photon energy range considered (10 keV–10 MeV), excellent agreement is observed between the two Monte Carlo codes for all tissues investigated. For blood irradiation (figure 3), SAFs for the alveolar interstitium, liver, red bone marrow, and small intestine stem cells exhibit consistent trends between the two Monte Carlo codes with differences not exceeding 5.3% across all energies. Unlike the other source-target combinations in figure 3, SAF(R-marrow ← Blood) initially decreases and then increases at low photon energies. At very low energies, the dose is dominated by photons emitted from blood within the marrow cavity. As photon energy increases, a fraction of these photons escapes the marrow, reducing the local energy deposition. In contrast, photons emitted from blood outside the marrow cavity contribute negligibly to the red bone marrow at very low energies because they are strongly attenuated by the surrounding dense cortical bone. As photon energy increases further (approximately 30 keV), these photons increasingly penetrate the cortical bone and deposit their energies to the red bone marrow, resulting in the slight increase in SAF. Similarly, for liver irradiation (figure 4), SAFs for the liver, right colon stem cells, kidneys, and endosteal surface show near-uniform agreement across the photon energy range with differences not exceeding 13% across all energies and not exceeding 8.8% greater than 15 keV. The observed consistency between the two codes for multiple source-target combinations indicates a high level of numerical stability and supports the reliability of the dosimetric calculations derived from the MRCPs.

### 3.4. Age-dependent trends in SAFs

**Figure 5.**
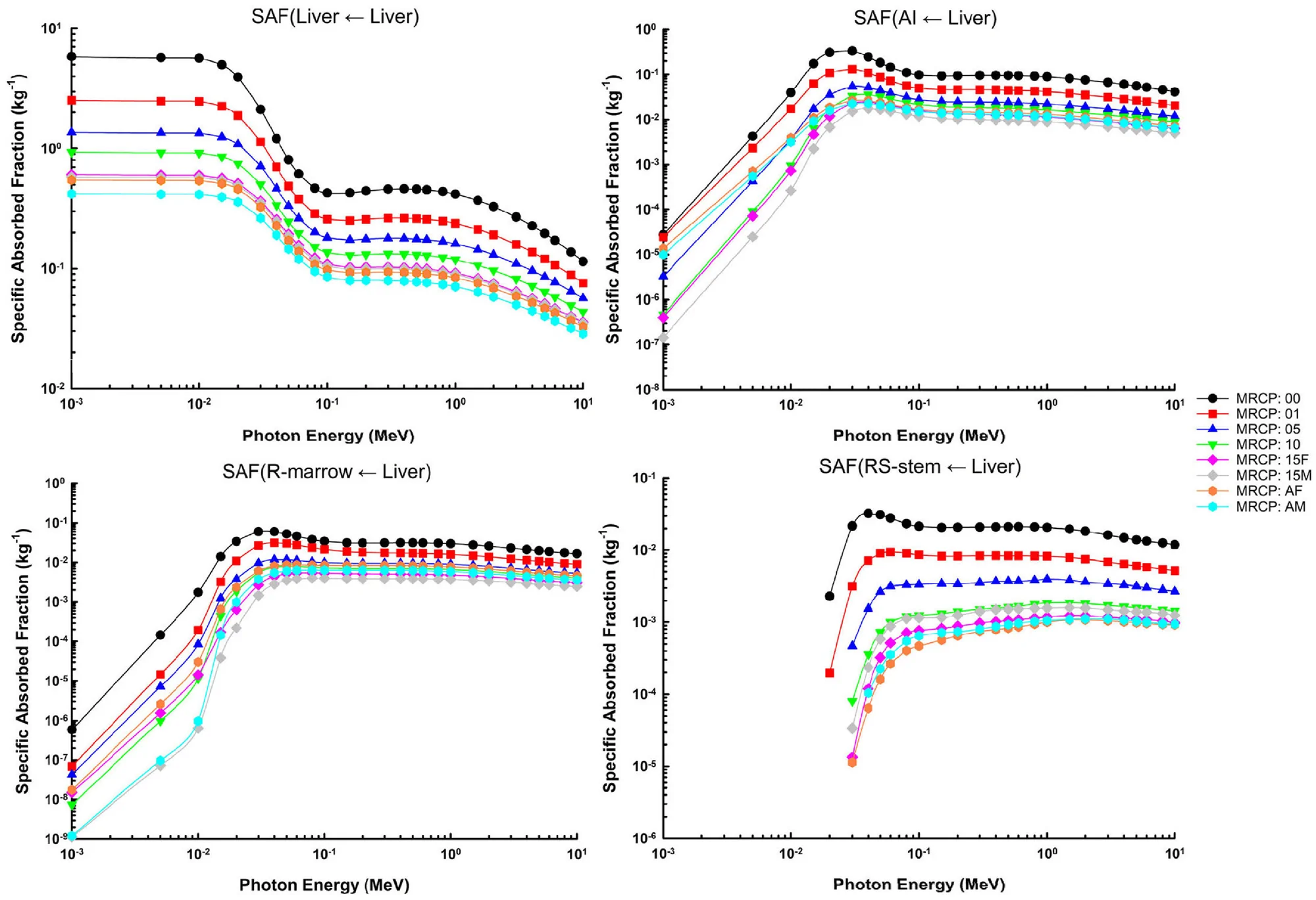
Specific absorbed fractions (SAFs) for photon exposures with the liver as the source and the liver, alveolar interstitium, red (active) bone marrow, and rectosigmoid stem cells as target tissues, calculated using the female newborn, 1-, 5-, and 10 yr old MRCPs, as well as male and female 15 yr old and adult MRCPs.

**Figure 6.**
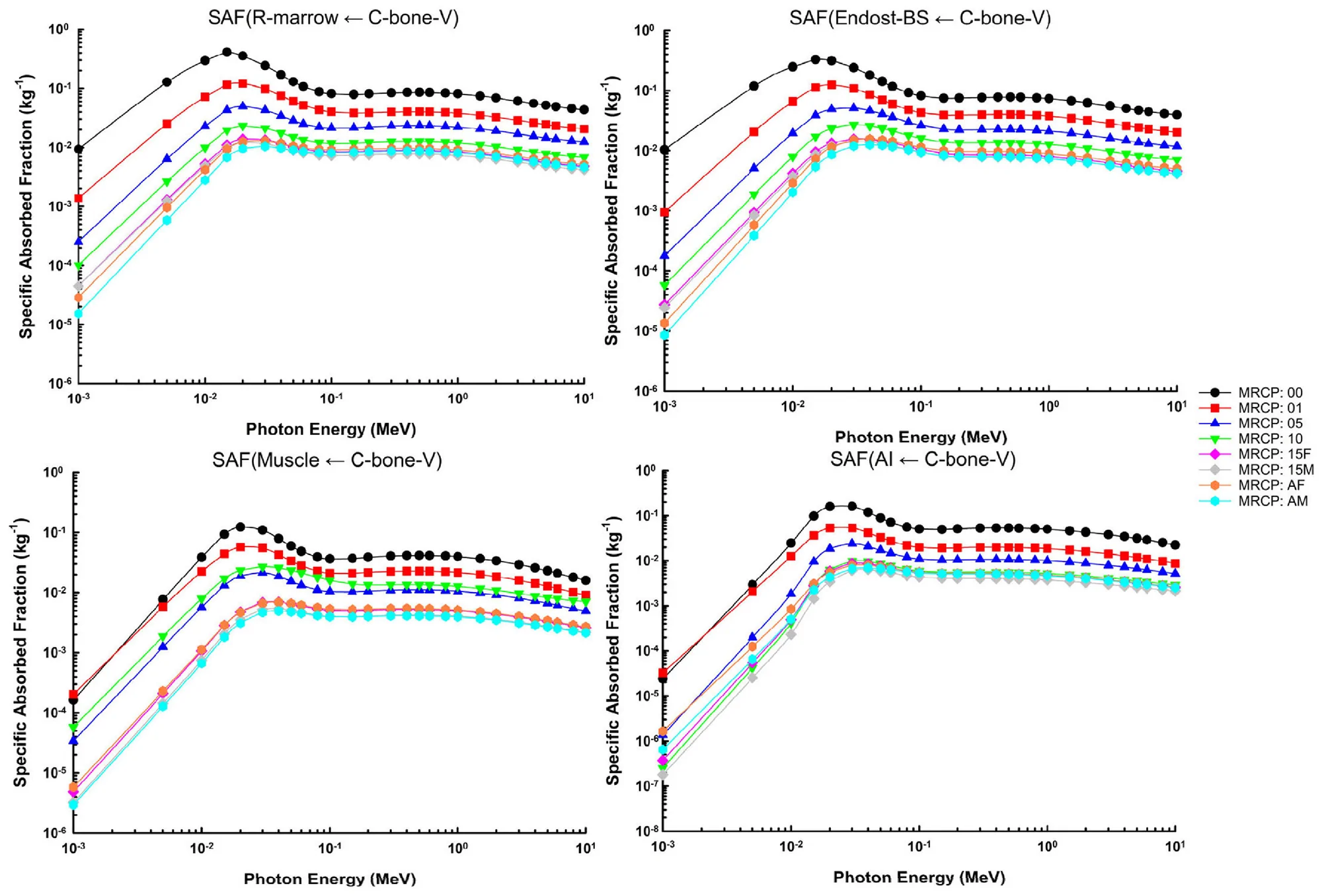
Specific absorbed fractions (SAFs) for photon exposures with cortical bone volume as the source and the red (active) bone marrow, endosteal surface, muscle, and alveolar interstitium as target tissues, calculated using the female newborn, 1-, 5-, and 10 yr old MRCPs, as well as male and female 15 yr old and adult MRCPs.

Figures 5 and 6 present SAFs for multiple source-target combinations calculated using the ICRP family of MRCPs across all available age groups. SAF results for the newborn, 1 yr old, 5 yr old, and 10 yr old models are shown for the female phantom only due to the male and female pediatric phantoms having similar anatomies, which in turn make the SAFs similar between the sexes at these ages. Across figures 5 and 6, consistent age-dependent trends are observed. Younger phantoms exhibit systematically higher SAF values compared with older children and adults. This can be attributed to smaller target organ masses in the younger phantoms as well as reduced inter-organ distances between the sources and targets.

### 3.5. Comparison of SAFs with ICRP Publications 133 and 155

**Figure 7.**
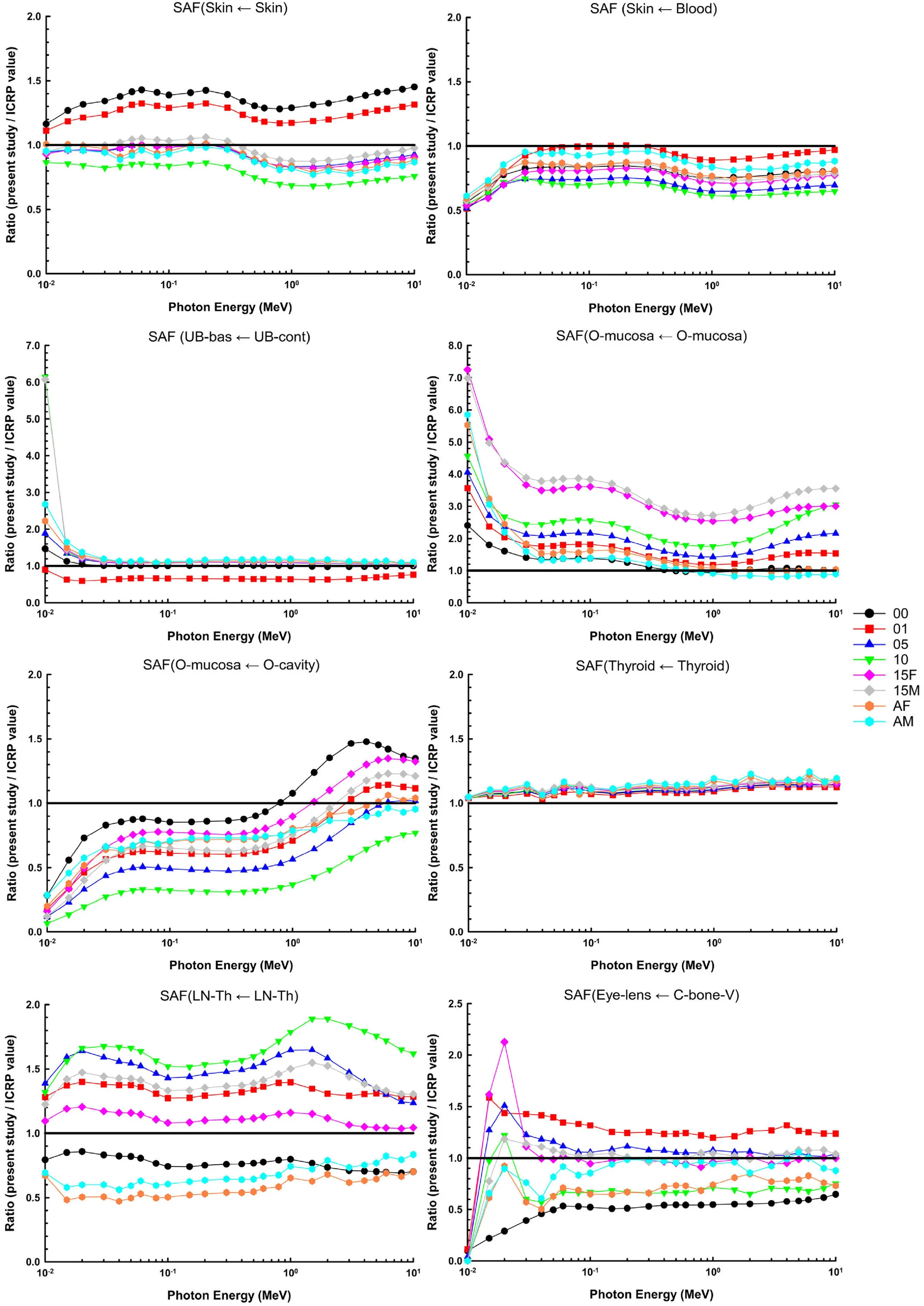
Ratios of specific absorbed fractions (SAFs) of mesh-type computational phantoms (MRCPs) to those from ICRP Publications 133 and 155, shown for source-target pairs involving organs and tissues whose geometry was modified during the development of the MRCPs from the voxel-based reference computational phantoms (VRCPs).

**Figure 8.**
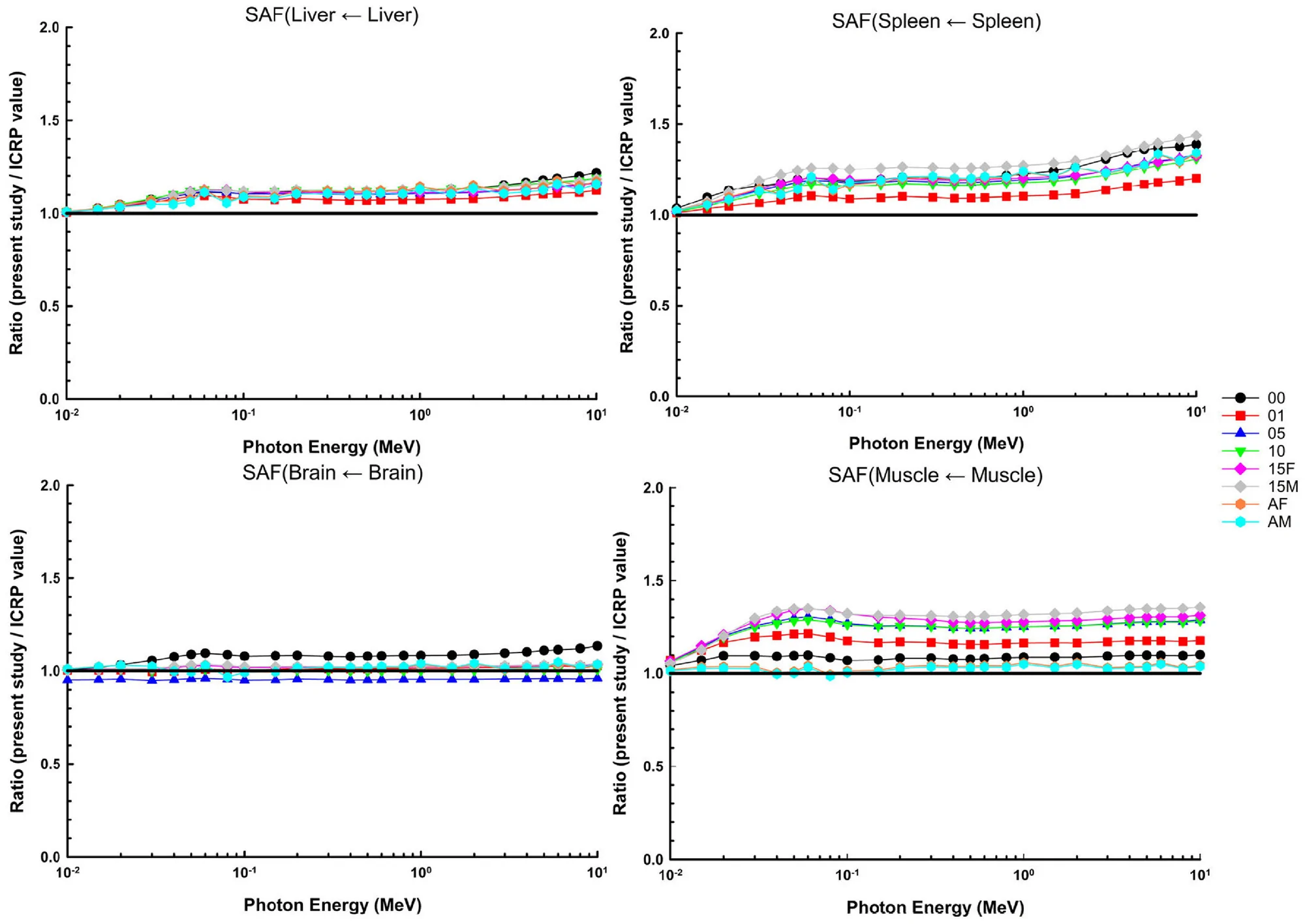
Ratios of specific absorbed fractions (SAFs) of mesh-type computational phantoms (MRCPs) to those from ICRP Publications 133 and 155, shown for source-target pairs involving organs with high blood volume fractions (liver and spleen) and low blood volume fractions (brain and muscle).

**Figure 9.**
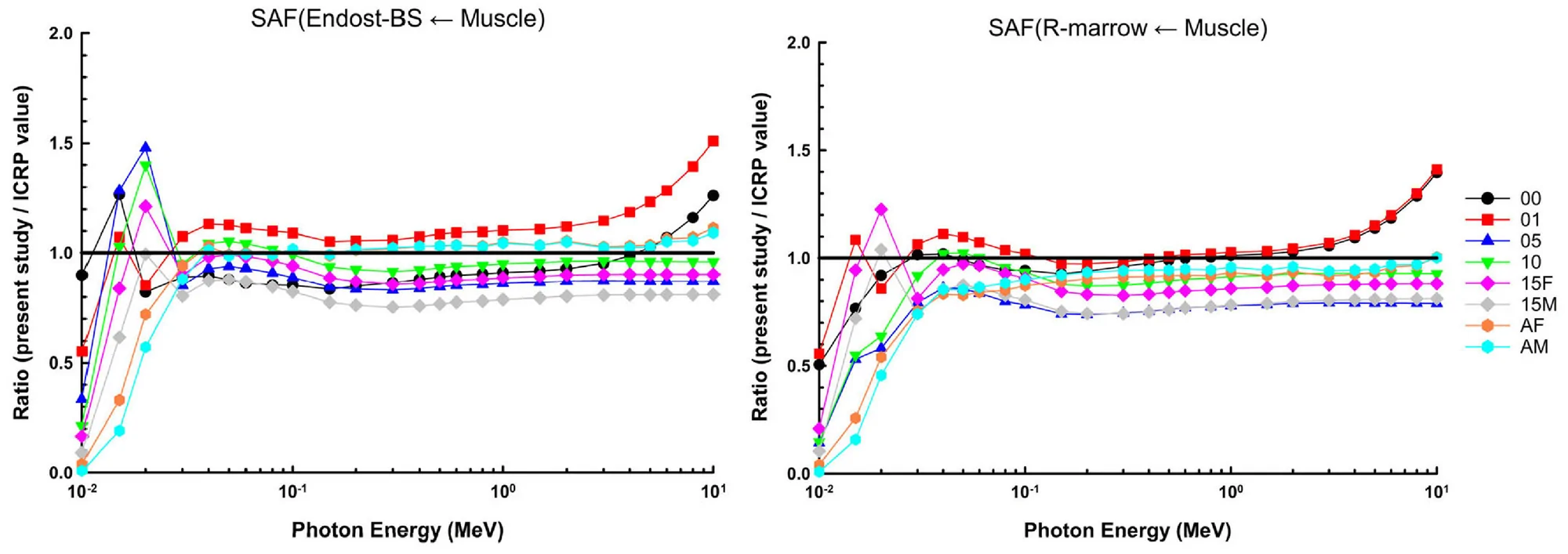
Ratios of specific absorbed fractions (SAFs) of mesh-type computational phantoms (MRCPs) with updated photon fluence-to-dose response functions (DRFs) to those from ICRP Publications 133 and 155, shown for endosteal surface and red (active) bone marrow targets from muscle sources.

**Figure 10.**
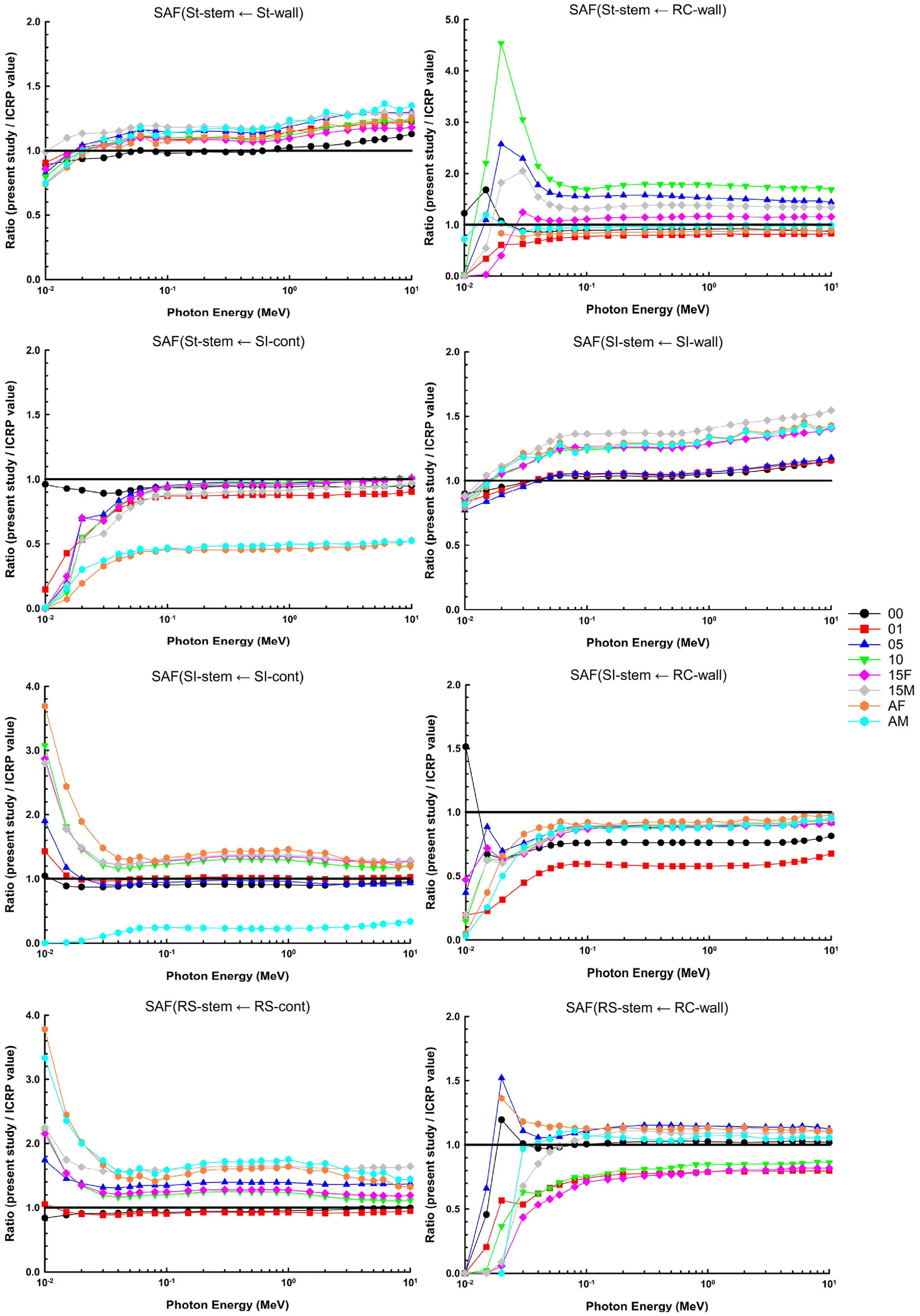
Ratios of specific absorbed fractions (SAFs) of mesh-type computational phantoms (MRCPs) to those from ICRP Publication 133 and 155, shown for source-target pairs involving the micrometer-scale target layers of the alimentary tract as explicitly defined in the MRCPs.

**Figure 11.**
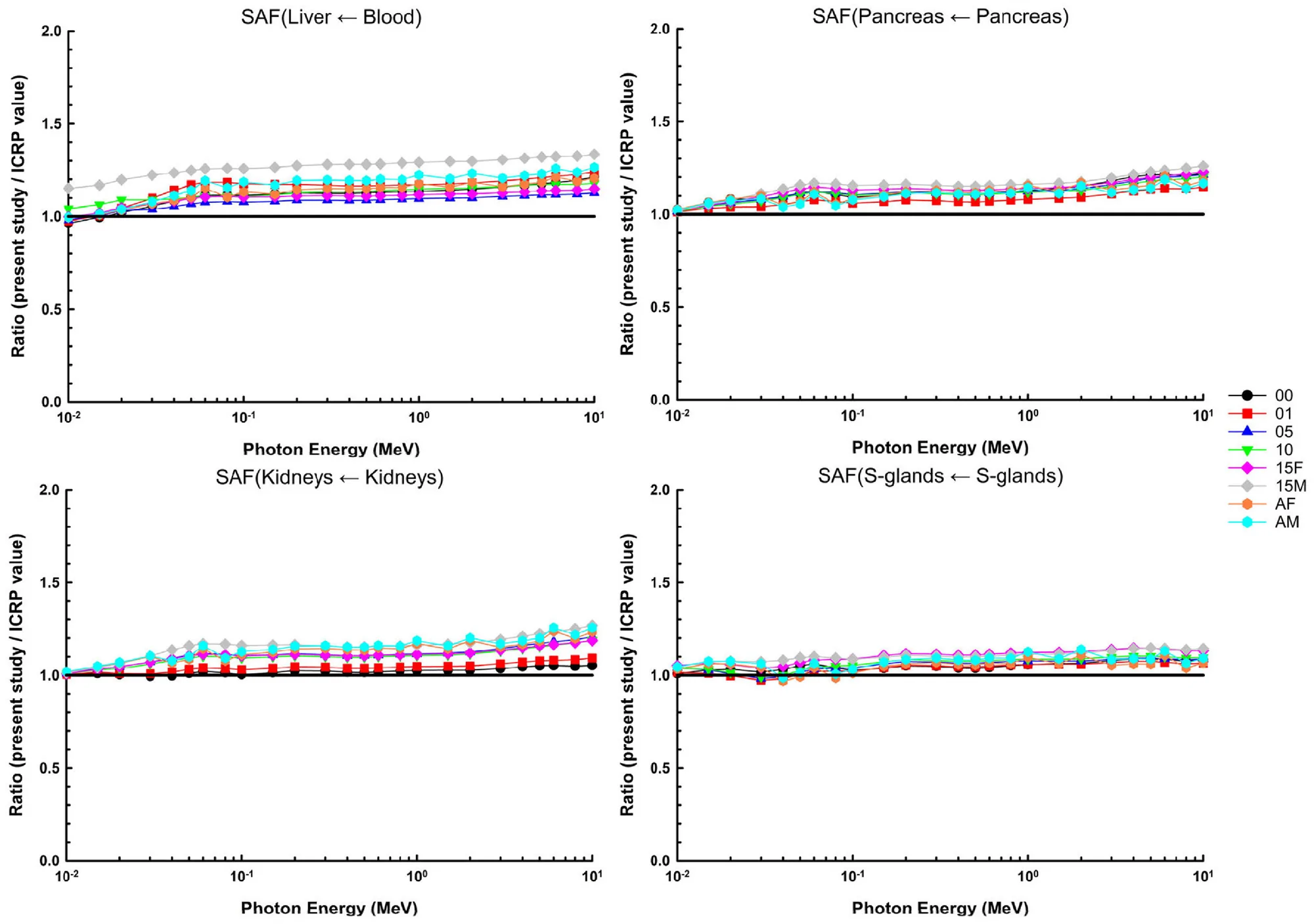
Ratios of specific absorbed fractions (SAFs) of mesh-type computational phantoms (MRCPs) to those from ICRP Publication 133 and 155, shown for source-target pairs that did not undergo substantial revisions in the creation of the MRCPs.

Figures 7–11 present ratios of SAFs between this study and those reported by the ICRP (ICRP 2016a, 2023) for source-target combinations highlighting the influence of anatomical revisions to organs and tissues in the MRCPs relative to the VRCPs (figure 7), the impact of explicit inclusion of intra-organ blood content within the MRCPs (figure 8), changes due to updated photon DRFs (figure 9), the influence of explicitly modeling micrometer-scale thin target tissue layers within the HATM in the MRCPs (figure 10), and additional source-target combinations for organs and tissues that did not undergo substantial revisions during MRCP development (figure 11).

Differences in the low-energy region (less than 40 keV) primarily reflect the ICRP’s implementation of the method outlined by Schwarz *et al* (2021) for low-energy extrapolation in which statistically stable Monte Carlo results may have been replaced to enforce linear behavior. In contrast, the present study retains calculated SAF values when statistical uncertainties remain acceptably low following GPR smoothing. Additionally, discrepancies in the low-energy range were also likely influenced by anatomical and geometric differences between the MRCPs and VRCPs.

Differences are also observed for red bone marrow and endosteal surface targets for muscle sources. These arise largely from ICRP’s recent updates to photon DRFs, as well as geometric limitations in the VRCPs where cortical bone does not completely envelop spongiosa. Whether the spongiosa is fully enclosed by cortical bone or contains openings may influence not only cases in which red bone marrow is treated as the target, but also those in which red bone marrow serves as the source and other organs/tissues are considered as targets. Further, notable deviations occur for thin targets (e.g. stem cell and basal layers), which are not explicitly modeled in the VRCPs; as noted in ICRP Publications 133 and 155, surrogate tissues for thin target layers were used, whereas this work explicitly models these thin tissue layers.

For organs and tissues that did not undergo substantial anatomical changes during MRCP development, SAF ratios remain close to unity across nearly the entire photon energy range, indicating good overall agreement between the MRCPs and VRCPs for these source-target pairs.

**Figure 12.**
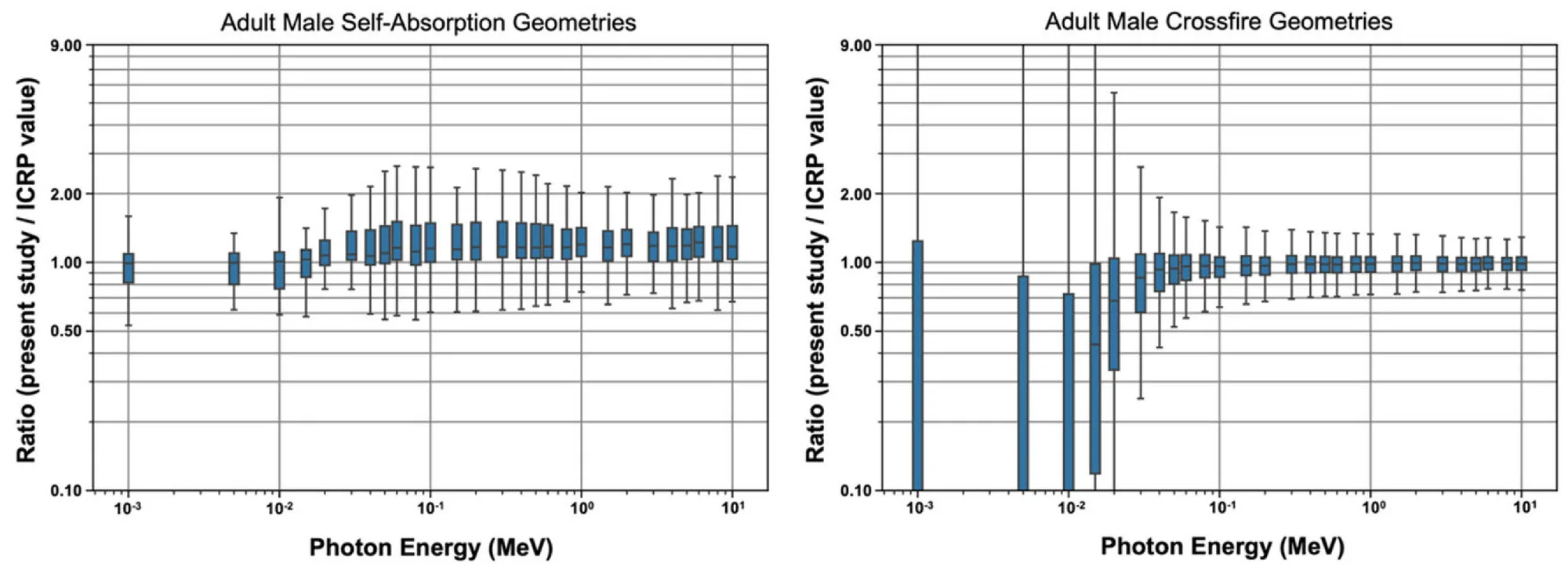
Boxplots of the distributions of ratios of specific absorbed fraction (SAF) values versus photon energy in the adult male for self-absorption geometries and crossfire geometries. The box defines the values of the first, second, and third quartiles of the values and the whiskers extend to 1.5 times the interquartile range.

Figure 12 visually describes the distribution of SAF ratios at each photon energy for the adult male. Source-target geometries have been split into two groups. Self-absorption geometries are those who have a non-zero SAF value at theoretical zero energy. In other words, they are those which have a target region which overlaps, at least in part, with the source region. Crossfire geometries are those geometries which do not overlap and therefore have a SAF of zero at theoretical zero energy. While the plots in figure 12 do not describe differences due to improvements or changes in individual geometries, they are useful for visualizing systematic changes.

As discussed earlier, in Publications 133 and 155 SAF scaling was required of self-absorption geometries to account for missing blood mass in the ICRP voxel phantoms. Publication 155 provides an overview of photon SAF scaling studies performed by Snyder (1971, 1971), Adams (1981), Petoussi–Henss *et al* (2007), and Wayson and Bolch (2018). As Snyder described, the simple linear mass scaling warranted for charged particles fails for photons at energies when Compton scattering predominates and the AF is below one-half. Publication 155 goes on to describe that ‘While an energy-dependent scaling such as described by Wayson and Bolch may improve photon SAFs >100 keV, in this work, the simpler linear mass scaling … was used for alpha particles, electrons, and photons.’

Had ICRP Publications 133 and 155 adopted a 2/3 power scaling for photon self-absorptions geometries, their SAF values would be larger. In figure 12 for self-absorption geometries, we observe values from the mesh phantoms where no scaling is required that are generally consistent with the linearly scaled SAFs in Publication 133 at the lowest energies (up to around 20 keV). But at 30 keV the mesh phantom SAFs begin to exceed their counterparts in Publication 133 and consistently do so through the highest photon energy.

For crossfire geometries in figure 12 (for which no scaling was needed in the prior ICRP work), the center of the distributions aligns very well with a ratio of 1.00 suggesting general agreement between 133 and the present work. Disagreement only exists at the lowest photon energies due in large part to the previously described low-energy extrapolation method. We further note that for crossfire geometries, these low-energy photons SAFs have small values and are frequently negligible contributors to internal doses.

## 4. Conclusion

In the present study, we produced a comprehensive set of photon SAFs using the ICRP adult and pediatric MRCPs using the PHITS Monte Carlo code. To the authors’ knowledge, this is the first study to provide complete photon SAF datasets for the full ICRP MRCP family, spanning all reference individuals from newborn to adult, covering all source regions and energy points defined within the ICRP dosimetric framework. When looking at the population of source-target geometries we observed generally good agreement with ICRP Publications 133 and 155 for crossfire geometries. For self-absorption geometries, good agreement was also observed at the lowest photon energies (<30 keV), but at higher energies we observed differences consistent with what would be expected when comparing to linearly scaled SAFs at energies where Compton scattering is significant and AFs are below one-half. Additional differences can be attributed to several factors, including differences in low-energy extrapolation methodology, anatomical and geometric revisions between the MRCPs and VRCPs, updates to photon DRFs, and the explicit modeling of thin target tissue layers in the MRCPs. The photon SAFs also demonstrated consistent age-dependent trends, with younger reference individuals exhibiting higher SAF values reflecting smaller organ masses and reduced inter-organ distances. Collectively, the present photon SAF datasets represent a significant advancement in reference dosimetric data and provide a foundation for future updates to reference ICRP dose coefficients.

## Data Availability

The data that support the findings of this study are openly available at the following URL/DOI: https://github.com/wsmither17/PMB-MRCP_photon_SAFs.git.
